# Population-based analysis of radiation-induced gliomas after cranial radiotherapy for childhood cancers

**DOI:** 10.1093/noajnl/vdac159

**Published:** 2022-10-03

**Authors:** Jacob B Leary, Amy Anderson-Mellies, Adam L Green

**Affiliations:** University of Colorado School of Medicine, Aurora, Colorado, USA; University of Colorado Cancer Center, Aurora, Colorado, USA; University of Colorado School of Medicine, Aurora, Colorado, USA; University of Colorado Cancer Center, Aurora, Colorado, USA; Morgan Adams Foundation Pediatric Brain Tumor Research Program, Children’s Hospital Colorado, Aurora, Colorado, USA

**Keywords:** epidemiology, radiation-induced glioma, radiotherapy, secondary tumor

## Abstract

**Background:**

Cranial radiotherapy (RT) used for pediatric CNS cancers and leukemias carries a risk of secondary CNS malignancies, including radiation-induced gliomas (RIG). Our aim was to characterize the epidemiology of RIG.

**Methods:**

This retrospective study used SEER data (1975–2016). Cohort 1 included patients diagnosed with glioma as a second malignancy ≥2 years after receiving treatment for a first malignancy diagnosed at 0–19 years, either a primary CNS tumor (1a, *n* = 57) or leukemia (1b, *n* = 20). Cohort 2 included patients who received RT for a pediatric CNS tumor and died of presumed progressive disease >7 years after diagnosis, since previous studies have documented many missed RIGs in this group (*n* = 296). Controls (*n* = 10 687) included all other patients ages 0–19 years who received RT for a first CNS tumor or leukemia.

**Results:**

For Cohort 1, 0.77% of patients receiving cranial RT developed RIG. 3.39% of patients receiving cranial RT for primary CNS tumors fell in cohort 2. Median latency to RIG diagnosis was 11.1 years and was significantly shorter for cohort 1b than 1a. Median OS for cohort 1 was 9.0 months. Receiving surgery, radiation, or chemotherapy were all associated with a nonstatistically significant improvement in OS (*P* .1–.2). A total of 1.8% of all brain tumor deaths fell in cohort 1, with 7.9% in cohort 2.

**Conclusion:**

A total of 1%–4% of patients undergoing cranial RT for pediatric cancers later developed RIG, which can occur 3–35 years after RT. Given the substantial and likely underestimated impact on overall CNS tumor mortality, RIG is deserving of increased attention in preclinical and clinical studies.

**Key Points**

- In total, 1%–4% of patients undergoing cranial RT for pediatric cancers went on to develop RIG.
- RIG may occur up to 30 years after cranial RT, warranting extended follow-up of exposed patients.
- A total of 2%–10% of pediatric brain tumor deaths are associated with RIG.

**Importance of the Study**

This is the first population-based study of the epidemiology of pediatric radiation-induced glioma (RIG), an incurable result of radiation therapy (RT) used for pediatric CNS tumors and leukemias and an understudied cause of pediatric CNS tumor death. By studying groups of probable/definite and possible RIGs, we characterize the range of incidence rates and impact on pediatric cancer mortality. We also demonstrate that RIG can occur decades after RT, demonstrating the need for extended follow-up. Finally, we show that, while standard treatments may have an impact on survival, no therapy has shown a significant extension of the devastatingly short median survival, highlighting a clear need for more study toward therapeutic advances.

CNS tumors are the second most common pediatric malignancy but the leading cause of cancer-related death in young patients. Of these, roughly 10% are classified as pediatric high-grade gliomas (pHGG), but these account for 40% of CNS tumor-related deaths.^1^ Given the potentially devastating consequences of these malignancies in terms of significant early-life morbidity and mortality, it is crucial to better understand the epidemiology of all subtypes of the disease.

The current standard-of-care treatment for pHGG continues to be maximal safe resection followed by focal radiation therapy (RT). External-beam RT, which involves a radiation source of protons or photons located outside a patient’s body, is also commonly employed as an adjunct therapy to treat embryonal tumors such as medulloblastoma, as well as ependymoma, and other CNS tumors.^2^ Often, a combination of partial resection, RT, and chemotherapy is used to maximize the likelihood of total eradication or at minimum, to increase the duration of progression-free survival and overall survival.^3,4^ In pediatric leukemias, cranial RT has been used as preventive therapy for leptomeningeal spread and in cases of CNS disease at diagnosis; its overall use and doses given have decreased over time due to concerns about neurodevelopmental outcomes and increased use of intrathecal chemotherapy.^5^

RT exerts its effects via DNA damage, specifically through double-strand breaks. The efficacy of this treatment is highly dependent on the amount of DNA damage induced.^6^ A more severe degree of DNA damage is more difficult for tumor cell DNA damage response (DDR) mechanisms to repair, increasing overall tumor cell death and thus more significantly decreasing tumor burden. However, DNA damage also occurs in healthy cells exposed to RT. Tremendous progress has been made over time to limit both dose and field of radiation exposure to minimize damage to healthy cells, which can lead to growth impairment, cognitive deficits, and secondary malignancies.^7^ Despite these strides in RT technique, some degree of inherent risk remains, especially when a larger tumor or larger portion of the brain requires irradiation.

Radiation-induced glioma/glioblastoma (RIG) is a high-grade secondary tumor arising in the CNS in regions previously irradiated, with or without systemic chemotherapy carrying the potential for additional DNA damage, due to a histologically distinct prior malignancy at an earlier age in childhood.^8^ These characteristics meet with those developed by Cahan *et al.* for radiation-induced solid tumors in 1948: Occurrence within the original radiation field, only occurring after a latency period following radiation, histological distinctness from the original tumor, and with no cancer predisposition syndrome present.^9^ RIGs are thought to occur most commonly between 5 and 15 years following treatment for the primary malignancy and are poorly responsive to antitumor therapies, including RT.^10^ RIG occurs most commonly following treatment for ependymoma, medulloblastoma, and leukemia.^11^ Prognosis for RIG is universally poor and often worse than other pHGG, given that preclinical models have been difficult to develop, and no dedicated clinical trials have been conducted. As a result, treatment for RIG is far less standardized than for other subtypes of pHGG, and though resection similarly offers a longer duration of OS,^12^ outcomes remain largely grim. It is known that RIG often has a more homogeneous profile of mutations vs *de novo* pHGG, with more overlap and clustering of genetic signatures in RIG than in other pHGG. Whereas *IDH* and *H3K27M* mutations are common in other pHGG subtypes, they are extremely rare in RIG.^13,14^

Whereas molecular characteristics are beginning to be elucidated for RIG, little is known about the epidemiology of these secondary tumors. The purpose of the present study was to provide a characterization of patients with RIG derived from the surveillance, epidemiology, and end results (SEER) Program registry, focusing on descriptive features of these tumors and the patients that they affect. Specifically, we aimed to define the true incidence of RIG including patterns of change in incidence over time, response to various therapeutic modalities, risk factors for RIG development, and the timeframe during which RIG development typically occurs following RT. We also sought to determine factors influencing the overall survival (OS) of these tumors.

## Methods

### Study Design and Data Collection

This is a retrospective case-control study. Patient-level data were obtained from the SEER Program of the National Cancer Institute, a collection of population-based cancer registries throughout the United States. The years included in this study (1975–2016) encompass varying groupings of participating registries with population coverage ranging from 9% in 1975–1991 (SEER-9 registries) to 28% during 2000–2016 (SEER-18 registries). Patient demographics and cancer histories were abstracted from the SEER-18 Registries Custom Data, November 2018 Submission using SEER*Stat Version 8.3.6. Available information on patient age and tumor characteristics at the time of diagnosis and first course of treatment was collected for each tumor in a patient’s history, as well as vital status, cause of death, and survival time.^15^

### Defining RIG

Constructing our cohort of patients diagnosed with RIG (cohort 1) began with selecting those who had been diagnosed with a Grade III/IV or ungraded glioma as a second primary malignancy at least 2 years after receiving beam radiation and/or chemotherapy for a first primary malignancy diagnosed at age 0–19 years, and had a history of no more than two primary malignancies (*n* = 124). The only patients included who had potentially undergone chemotherapy alone were patients with leukemia whose beam radiation treatment status was unknown; we elected to include these patients as possible RIG cases since prior radiation status was defined for very few leukemia patients, which appeared to be an issue in SEER specific to this disease group. Second primary glioma subtypes eligible for inclusion were as follows: Anaplastic astrocytoma, diffuse astrocytoma, glioblastoma, oligodendroglioma, anaplastic oligodendroglioma, pilocytic astrocytoma, unique astrocytoma variants, mixed glioma, astrocytoma not otherwise specified (NOS), glioma NOS, benign and malignant neuronal/glial, neuronal, and mixed tumors; and unspecified CNS neoplasms. Patients with more than two primary malignancies were excluded, as this may be indicative of a tumor predisposition syndrome. Patient histories were then manually reviewed for primary tumor type/treatment and secondary tumor type. We excluded 44 patients whose first primary malignancy was neither CNS nor leukemia and patients whose second malignancy developed at a site presumed to be outside of the initial RT field. We included patients with an original diagnosis of glioma if their later tumor was classified as secondary in SEER. One patient with precursor T-cell lymphoblastic lymphoma affecting lymph nodes in multiple regions, and one patient with osteosarcoma of the mandible were excluded as their beam RT treatment status was no/unknown. We also excluded 3 additional patients with a first primary CNS malignancy whose beam radiation treatment status was no/unknown (*n* = 1) or who received non-beam RT (*n* = 2; 1 received radioactive implant brachytherapy and the other received RT, NOS). Cohort 1 was then further divided into those who were confirmed to have been treated with beam radiation for their first malignancy (cohort 1a; *n* = 57), and those patients with leukemia whose radiation treatment status were unknown for their first malignancy (cohort 1b; *n* = 20).

As the second cohort of possible undiagnosed RIG (cohort 2), we included any other patient aged 0–19 years who received beam radiation for a first primary CNS tumor whose death occurred 7 or more years after diagnosis (10 or more years for ependymomas, as these, are known to have late true recurrences) and was attributed to their cancer. This cohort was included because it is now known that primary tumor recurrences this late after diagnosis are very rare, and many of these tumors may actually be RIGs that were either never biopsied or pathologically misclassified.^16,17^ This cohort was further divided into non-glioma (2a; *n* = 139) and glioma (2b; *n* = 157) as first malignancy to allow for distinction in the case that some of cohort 2b patients may have had a rare late recurrence rather than a RIG.

The control population for all cohorts included all other patients aged 0–19 years who received beam radiation for a first primary CNS tumor or leukemia and who did not fit the inclusion criteria for cohorts 1 or 2 (control; n = 10 687).

### Outcomes of Interest

The primary outcomes of interest included demographic and tumor-specific characteristics for the RIG cohorts compared to controls, including age at initial diagnosis, sex, race, ethnicity, initial tumor type, and treatment type for first primary malignancy. Additionally, we sought to identify the incidence of RIG to characterize the overall risk of developing these tumors following treatment for the original pediatric malignancy. Other primary outcomes of interest included lag time between diagnosis of the first primary CNS tumor or leukemia and development of RIG, and overall survival (OS) for patients who developed RIG, both measured in months.

Secondary outcomes of interest focused on the incidence of RIG development broken down by treatment type for the first primary CNS tumor or leukemia. We also sought to evaluate overall survival based on treatment type for RIG.

### Statistical Analysis

All statistical analyses were performed using SAS software, Version 9.4 (SAS Institute Inc., Cary, NC), with significance defined as *P*-value <.05. All patients included in analyses had data available for all variables of interest in the SEER registries. The cohorts are used collectively to describe the occurrence of RIGs. Each cohort was compared to the control population on distributions of age at initial diagnosis, sex, race, ethnicity, initial tumor type, and treatment of initial tumor using Chi-square and Fisher’s exact tests. The proportion of CNS tumor deaths potentially attributable to RIGs was also estimated. Kaplan–Meier plots were used to visualize lag time between initial and RIG diagnoses, as well as to evaluate the cumulative incidence of RIG and OS following RIG diagnosis. Univariate effects of treatment modality of RIG on survival were evaluated with the log-rank test.

### Ethics Statement

This study was exempt from institutional review board or ethics committee review due to its population-based nature using deidentified cancer registry data only.

## Results

Patient demographic and tumor-specific characteristics, including comparisons between groups, are displayed in Table 1. In terms of initial primary malignancies, cohort 1a contained a predominance of medulloblastomas (38.6%), gliomas (26.3%), and leukemias (17.5%). Cohort 1b was composed entirely of leukemias treated with chemotherapy and with an unknown RT treatment status. Cohorts 2a and 2b were comprised of patients with secondary tumors that were considered possible RIGs, but were less clearly attributable to RT. In cohort 2a, the most common original diagnoses were medulloblastoma (54.7%), PNET/pineal gland tumor (13.7%), germ cell tumor (13.0%), and ependymoma (12.2%). Cohort 2b was entirely composed of gliomas. See Table 1 for complete details for each group.

**Table 1.** Patient Demographic and Tumor-Specific Characteristics of RIG/Possible RIG Cohorts vs Control Cohort

Characteristic	Cohort 1a			Cohort 1b			Cohort 2a			Cohort 2b			Control	
	*N*	% with attribute	*P* ^1^	*N*	% with attribute	*P* ^1^	*N*	% with attribute	*P* ^1^	N	% with attribute	*P* ^1^	*N*	% with attribute
Overall	57	0.52		20	0.18		139	1.26		157	1.42		10 687	96.60
*Age at original diagnosis*			.610			.010			.790			.002		
<1 year	1	1.75		1	5.00		1	0.72		7	4.46		213	1.99
1–4 years	13	22.81		11	55.00		32	23.02		27	17.20		2399	22.45
5–9 years	21	36.84		4	20.00		44	31.65		40	25.48		3113	29.13
10–14 years	14	24.56		3	15.00		31	22.30		33	21.02		2716	25.41
15–19 years	8	14.04		1	5.00		31	22.30		50	31.85		2246	21.02
*Sex*			.091			.706			.633			.205		
Female	17	29.82		9	45.00		54	38.85		72	45.86		4366	40.85
Male	40	70.18		11	55.00		85	61.15		85	54.14		6321	59.15
*Race*			.218			.343			.206			.319		
White	42	73.68		15	75.00		104	74.82		127	80.89		8498	79.52
Black	5	8.77		1	5.00		23	16.55		19	12.10		1149	10.75
AI/AN	0	0.00		0	0.00		2	1.44		3	1.91		96	0.90
Asian/PI	10	17.54		4	20.00		10	7.19		8	5.10		904	8.46
Unknown	0	0.00		0	0.00		0	0.00		0	0.00		40	0.37
*Ethnicity*			.011			.754			.074			<.001		
Non-Hispanic	52	91.23		16	80.00		116	83.45		145	92.36		8235	77.06
Hispanic	5	8.77		4	20.00		23	16.55		12	7.64		2452	22.94
*Original diagnosis*			<.001			—			—			—		
Glioma	15	26.32		0	0.00		0	0.00	—	157	100.00	—	3314	31.01
Ependymoma	3	5.26		0	0.00		17	12.23	—	0	0.00	—	821	7.68
Choroid plexus tumor	1	1.75		0	0.00		2	1.44	—	0	0.00	—	18	0.17
Medulloblastoma	22	38.60		0	0.00		76	54.68	—	0	0.00	—	1624	15.20
PNET/Pineal	1	1.75		0	0.00		19	13.67	—	0	0.00	—	546	5.11
ATRT	1	1.75		0	0.00		1	0.72	—	0	0.00	—	112	1.05
Germ cell tumor	4	7.02		0	0.00		18	12.95	—	0	0.00	—	618	5.78
Teratoma	0	0.00		0	0.00		0	0.00	—	0	0.00	—	34	0.32
Other/unspecified CNS	0	0.00		0	0.00		6	4.32	—	0	0.00	—	183	1.71
Leukemia	10	17.54		20	100.00		0	0.00	—	0	0.00	—	3417	31.97
*Treatment of original tumor*			.089			—			.111			<.001		
Beam radiation, no/unknown chemotherapy	22	38.60		—	—		48	34.53		122	77.71		3035	28.40
Beam radiation + chemotherapy	35	61.40		—	—		91	65.47		35	22.29		7652	71.60
Chemotherapy, no/unknown beam radiation	—	—		20	100.00		—	—		—	—		—	—
*Histology of RIG*			—			—			—		—	—	—	—
Diffuse astrocytoma (protoplasma, fibrillary)	1	1.75		0	0.00		—	—		—	—		—	—
Anaplastic astrocytoma	8	14.04		3	15.00		—	—		—	—		—	—
Glioblastoma	24	42.11		13	65.00		—	—		—	—		—	—
Pilocytic astrocytoma	0	0.00		0	0.00		—	—		—	—		—	—
Oligodendroglioma	1	1.75		0	0.00		—	—		—	—		—	—
Anaplastic oligodendroglioma	1	1.75		1	5.00		—	—		—	—		—	—
Mixed glioma	0	0.00		1	5.00		—	—		—	—		—	—
Astrocytoma, NOS	6	10.53		0	0.00		—	—		—	—		—	—
Glioma, NOS	13	22.81		1	5.00		—	—		—	—	—	—	—
Benign and malignant neuronal/glial, neuronal and mixed	1	1.75		1	5.00		—	—		—	—	—	—	—
Neoplasm, unspecified, benign and malignant	2	3.51		0	0.00		—	—		—	—	—	—	—
*Year of Original Diagnosis* ^b^			—			—			—			—		
1975–1979	10	17.54		0	0.00		27	19.42		46	29.30		1039	9.72
1980–1984	10	17.54		0	0.00		15	10.79		41	26.11		858	8.03
1985–1991	6	10.53		4	20.00		17	12.23		30	19.11		989	9.25
1992–1999	9	15.79		5	25.00		34	24.46		22	14.01		1436	13.44
2000–2004	14	24.56		8	40.00		38	27.34		10	6.37		1928	18.04
2005–2009	7	12.28		2	10.00		8	5.76		8	5.10		1909	17.86
2010–2016	1	1.75		1	5.00		0	0.00		0	0.00		2528	23.65

^a^ *P*-value from Chi-square (or Fisher’s Exact for cells <5) test comparing cohort to control.

^b^The number of registries participating in SEER varies over the study period, with additional registries added in 1992 and 2000.

Abbreviation: RIG, radiation-induced glioma.

### Incidence

The cumulative incidence of developing RIG, using only Cohort 1a as events and excluding Cohort 1b from calculations, was 0.48% at 10 years, 0.87% at 15 years, 1.13% at 20 years, and 2.58% at 35 years (Supplementary Figure 1). RIG incidence data were further evaluated in 2 other ways. We first investigated the incidence of new RIG cases by year of original diagnosis, as a proportion of the total cases of new-onset first primary malignancies diagnosed in a given year that later went on to develop RIG. From 1975 to 2016, mean incidence per year in each cohort was as follows: Cohort 1 overall = 0.77% (range: 0%–2.65%); cohort 1a = 0.57% (range: 0%–2.65%), cohort 1b = 0.20% (0%–1.37%), cohort 2a = 1.47% (0%–3.57%), and cohort 2b = 1.92% (0%–6.67%). For both cohorts 1a and 1b, a trend toward decreasing incidence over time was observed via the 5-year moving average, and this was replicated when Cohort 1 was analyzed overall (Supplementary Figure 2A). The second method of evaluating RIG incidence involved determining the proportion of all CNS tumors diagnosed in a given year that were classified as RIG. From 1977 to 2016, the mean annual incidence of RIG using the cohort 1a definition was 0.034% (range: 0%–0.116%), while for cohort 1b this was 0.011% (range: 0%–0.057%). Combined, the mean annual incidence of RIG using the cohort 1 definition was 0.04% (range: 0%–0.17%). Using the 5-year moving average, a trend of increasing incidence over time was observed for cohorts 1a, 1b, and cohort 1 overall (Supplementary Figure 2B).

### Latency to RIG Diagnosis

**Figure 1.**
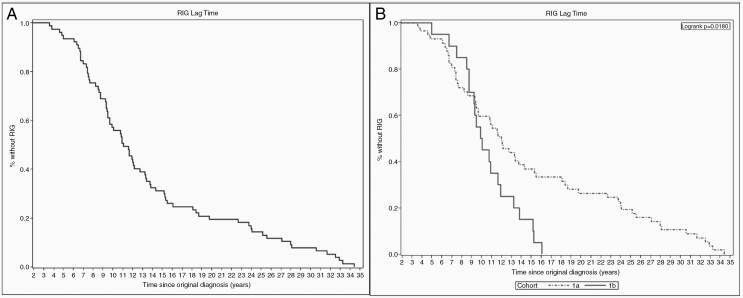
(**A**) Latency to RIG development for cohort 1 overall. The majority of patients developed RIG within the first 15 years post-original diagnosis, but 20% of patients developed RIG beyond this point. (**B**) Latency to RIG development, cohort 1a vs 1b. Patients in cohort 1b developed RIG significantly faster than those in cohort 1a. RIG = radiation-induced glioma.

**Figure 2.**
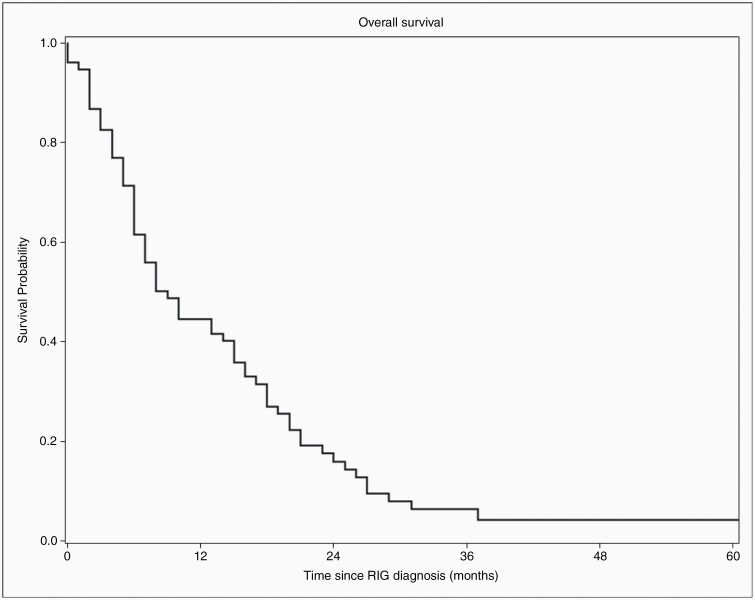
The median OS observed for cohort 1 was 9.0 months.

Latency period between original diagnosis and development of RIG is shown in Table 2. Overall, the median latency until RIG development for cohort 1 was 11.1 years (minimum = 3.58 years, maximum = 34.42 years; Figure 1). Cohort 1a had a significantly longer median latency to RIG diagnosis as compared with cohort 1b (12.0 years vs 10.0 years, *P* = .018; Figure 2).

**Table 2.** Latency Between Original Diagnosis and RIG Diagnosis, in Years

	Mean	SD	Median	Min	Max
Cohort 1	13.85	8.08	11.08	3.58	34.42
Cohort 1a	14.99	8.97	12.00	3.58	34.42
Cohort 1b	10.61	2.95	10.00	5.00	16.08

Abbreviation: RIG, radiation-induced glioma.

### Overall Survival

OS for cohort 1 is displayed in Figure 2. Median OS for patients in Cohort 1 was 9.0 months. One year post-RIG diagnosis, OS was 44.5% (95% CI = 32.8%–55.5%). Two-year OS was 15.9% (95% CI = 8.4%–25.7%), and 3-year OS was 6.4% (95% CI = 2.1%–14.1%). Over the course of the study period, 88% (50/57 patients) of patients in cohort 1a died from RIG, while the mortality rate for patients in cohort 1b was 80% (16/20 patients). In contrast, 43% (4626/10 687) of control patients died over the course of the study period. Table 3 provides detailed information regarding overall deaths by cohort and total deaths broken down by original diagnosis.

**Table 3.** Proportion of Deaths from RIG Overall and by Original Diagnosis

	Cohort 1a		Cohort 1b		Cohort 2a		Cohort 2b		Total deaths (Cohorts + Controls)
	N	% of deaths	N	% of deaths	N	% of deaths	N	% of deaths	
Overall	50	1.00	16	0.32	139	2.79	157	3.15	4988
Original diagnosis									
Glioma	13	0.55	0	0.00	0	0.00	157	6.69	2347
Ependymoma	3	1.09	0	0.00	17	6.18	0	0.00	275
Choroid plexus tumor	1	10.00	0	0.00	2	20.00	0	0.00	10
Medulloblastoma	20	3.45	0	0.00	76	13.13	0	0.00	579
PNET/Pineal	1	0.37	0	0.00	19	7.12	0	0.00	267
ATRT	1	2.33	0	0.00	1	2.33	0	0.00	43
Germ cell tumor	4	3.70	0	0.00	18	16.67	0	0.00	108
Teratoma	0	0.00	0	0.00	0	0.00	0	0.00	13
Other/unspecified CNS	0	0.00	0	0.00	6	8.33	0	0.00	72
Leukemia	7	0.55	16	1.26	0	0.00	0	0.00	1274

Abbreviation: PNET, primitive neuro-ectodermal tumor; RIG, radiation-induced glioma.

**Figure 3.**
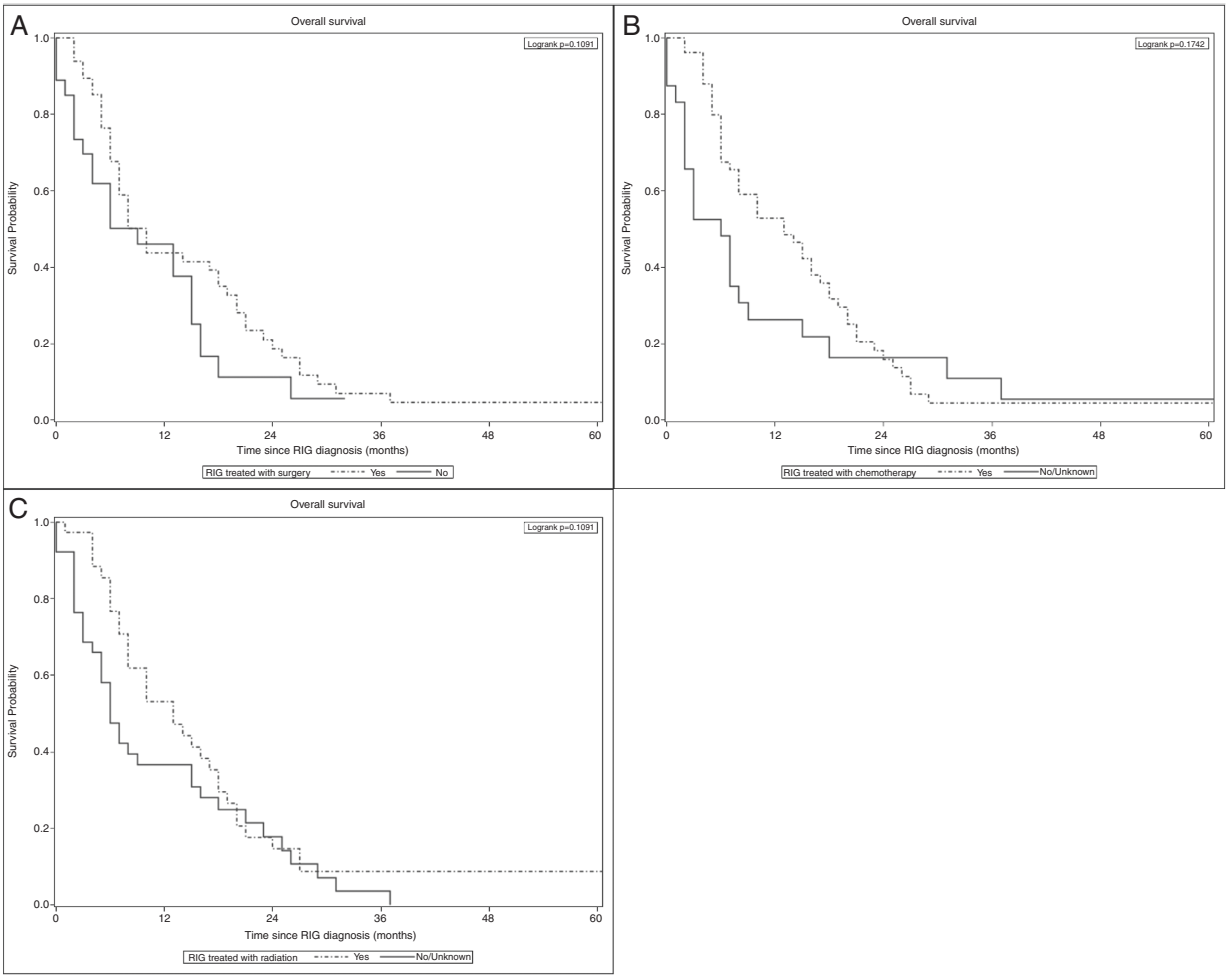
(**A**) OS with and without surgery for RIG. (**B**) OS with and without chemotherapy for RIG. (**C**) OS with and without radiation for RIG. No significant differences were observed in median OS between groups who did or did not receive treatment regardless of modality, including surgery, chemotherapy, and radiation.

We also compared OS between patients by treatment type received for RIG. When comparing the group who received surgery to those in the No/Unknown group (Figure 3A), no significant difference was observed in median OS (10.0 vs 9.0 months; *P* = .109). The group who received chemotherapy for RIG (Figure 3B) had a median OS of 13.0 months, which was not significantly different from the No/Unknown Chemotherapy group which had a median OS of 6.0 months (*P* = .174). Similarly, patients treated with radiation for RIG (Figure 3C) had a median OS of 13.0 months, which was not significantly longer than the No/Unknown Radiation group with a median OS of 6.0 months (*P* = 0.133).

## Discussion

Very little is understood about the epidemiology of RIG. The present study sought to characterize RIG using a population-based sample, including true incidence rates and changes in incidence over time, risk factors for RIG development, a timeline of RIG development following external-beam RT, and response to treatment with various modalities. We also aimed to better understand the median OS for RIG. Our work expands upon prior literature by showing that RIG occurred in a small but substantial proportion of those who underwent treatment for pediatric tumors affecting the CNS, with a mean incidence rate of 0.77% in cohort 1 by year of original diagnosis. We demonstrated that RIG may develop far beyond the RT treatment period, with a median lag time to RIG for cohort 1 of 11.1 years and a range that extended more than 34 years beyond external-beam RT exposure. RIG appears to be a highly lethal malignancy with a dismal prognosis, with median OS for cohort 1 of 9.0 months and only 6.4% of patients still living 3 years post-diagnosis. Additionally, we identified a group of patients comprising cohort 2 with possible undiagnosed RIG whose deaths occurred more than 7 years after their original diagnosis, providing a rationale for more in-depth pathologic analysis of patients’ tumors in these situations to accurately differentiate RIGs from recurrent tumors and allow accurate treatment.

Pediatric patients of all ages and races, male and female sex, and Hispanic and non-Hispanic ethnicities were observed to develop RIG in our sample. The age of patients in Cohort 1b was significantly different from controls, driven by the majority of cohort 1b developing leukemias between ages 1 and 4 years. Age in cohort 2b was also significantly different compared to controls, with children ages 15–19 years being more likely to develop glioma as their original primary malignancy. The most common original tumor diagnoses in confirmed RIG cases (cohort 1) were leukemias, followed by medulloblastomas and gliomas. Though these findings may be specific to our analysis and our methodology used to collect data within SEER, it should be noted that patients with these primary tumor types have previously been implicated as being among the most likely to develop RIG.^18^

Our work was conducted using data derived from the SEER-18 Registries Custom Data, covering the years 1975–2016. SEER contains detailed information regarding patient demographics, cancer diagnoses, and survival over time; however, some patient-level information was less reliably included, including specific treatments received. With RIG being defined by exposure to RT, this made it difficult to precisely determine the incidence of RIG, as some patients, especially those treated with cranial RT for leukemia, seemed to lack definitive data regarding whether they underwent external-beam RT. This necessitated further dividing cohort 1 into cohort 1a, comprised of those who were confirmed to have been treated with beam radiation for their first malignancy, and Cohort 1b, which included patients with leukemia whose RT treatment status was unknown. While we determined that the mean incidence of RIG by year of original diagnosis for cohort 1 was 0.77%, it is possible that this number could be closer to the mean incidence of 0.57% for cohort 1a if not all RIG patients with prior leukemia underwent RT.

Conversely, patients in cohort 2 represent cases of possible undiagnosed RIG, as they died more than 7 years following their first primary CNS malignancy diagnosis and treatment with external-beam RT. With primary CNS malignancies rarely recurring this late after initial treatment, it may be that at least some of these cases were not biopsied or were pathologically misclassified and would have fit molecular criteria for RIG. As we are unable to obtain samples and perform a central pathological review for possible RIG reclassification, it cannot be definitively confirmed which of these cases were indeed RIG. We included these patients to define the full potential scope of RIG and to highlight that the incidence numbers may be higher than reported here. These limitations within cohorts 1 and 2 found within SEER raise the importance of establishing a RIG-specific registry that would contain detailed information about these patients, their disease courses, and the pathology of their tumors, providing investigators with a more robust database for further studies. Our group has established such a registry for the purpose of better understanding this disease: The Pediatric RIG Registry (RIG-R).

Mean incidence of RIG by year of original diagnosis generally appeared to decline over time between 1975 and 2016. As we observed that the median latency to RIG was 11.1 years after diagnosis of first malignancy, it is likely that there has not yet been sufficient lag time for patients diagnosed and treated with RT in more recent years to go on to develop RIG, which may in part be driving the appearance of decreased case rates. There is hope that RT techniques that have improved targeting to limit normal tissue exposure may decrease secondary tumors, but when analyzing mean RIG incidence as a proportion of all CNS tumors diagnosed at any age in a given year, we found that incidence appeared to increase over time.

Our observed median OS of 9.0 months for patients with RIG portends a dismal prognosis for these individuals and is comparable to that of diffuse intrinsic pontine glioma, a pHGG with arguably the worst prognosis and which has proven to be exceedingly difficult to treat.^19,20^ Three years following diagnosis of RIG, only 6.4% of patients remained alive in our sample. These figures, while jarring, make sense in the context of our data that were unable to show significantly improved survival for patients who received surgery, RT, or chemotherapy. A prior report showed the benefit of resection of RIG over other modalities,^12^ but this previous study’s findings may have been influenced by very small sample size. Our findings are consistent with literature showing that RIG is poorly responsive to treatment^10^ and highlight the need for the development of better preclinical models and the initiation of clinical trials specifically targeting RIG.

Our work is largely concordant with the small but existing body of RIG literature. Incidence of RIG development after cranial RT has been estimated to occur in ~0.5%–3% of patients receiving cranial RT^10,21^ after a median latency period of 9–15 years,^22,23^ with a median OS of 9–11 months and 2-year survival rate of approximately 20%.^18,22^ While these retrospective cohort and systematic review data provide an important foundation for RIG epidemiology, we expand upon these here by providing annual incidence rates over 4 decades both by year of the original diagnosis and as a proportion of all CNS tumors diagnosed in a given year. This study is also the first of its kind to use a large, population-based dataset in an attempt to better characterize the incidence and impact of RIG with greater population representation than prior studies. A notable finding was that RIG development occurred as late as ~34 years after the original diagnosis in our sample, indicating the need for clinicians to be aware of the potential for RIG many years after RT exposure. This may have implications for long-term follow-up. It is also interesting to note the significantly shorter median latency to RIG for Cohort 1b compared to cohort 1a in our sample, which may be attributable to the long-term chemotherapy also received by these patients.

There are several limitations to our work. In this population-based study, we are obviously unable to apply molecular pathology techniques increasingly being used to give further evidence that a tumor is truly radiation-induced. Using data from SEER registries spanning 1975–2016, only 9% of the population was represented within registry data from 1975 to 1991, while approximately 28% of the population was covered between 2000 and 2016. This may limit the generalizability of these findings to the larger US population outside of geographic areas covered within these registries. Patients who were diagnosed more recently had shorter follow-ups and thus may misleadingly reflect a lower incidence of RIG. Inherent to the SEER database, it is possible that patient demographics such as race and ethnicity were misclassified, and a substantial number of patients had limited treatment history listed. The location/field of RT received is not available in SEER, which limits the ability to ensure the tumor was in the RT field. Information on chemotherapy is often incomplete, with one SEER category that includes patients known not to have received chemotherapy and whose chemotherapy history is unknown. We also opted to include only patients who received external-beam RT, which excluded patients who may have developed RIG as a result of other radiation treatment techniques and may underestimate the overall incidence of RIG.

## Conclusion

Using a large, population-based sample of pediatric patients with tumors affecting the CNS within the SEER registries, we characterized the annual incidence of RIG by year of the original diagnosis and as a proportion of all CNS tumors diagnosed at any age in a given year between 1975–2016. We found incidence rates concordant with existing literature and observed median latency to RIG that was beyond the previously established understanding of the timeframe within which RIG usually occurs. This suggests that clinicians should be aware of the potential need for following patients who underwent cranial RT as children beyond the first 15 years after treatment. We also found that RIG carries a very poor prognosis, with a median OS of 9.0 months and no particularly effective treatment options currently available. Focused effort should be made toward developing better preclinical models of RIG and conducting translational and clinical studies of therapies specifically targeted at this treatment-resistant tumor subtype. Establishment of a national registry of RIG patients and tumor pathology samples is an important first step toward this effort.

## Supplementary Material

vdac159_suppl_Supplementary_Data

vdac159_suppl_Supplementary_Figures
