# Supplementary material for: Population-based analysis of radiation-induced gliomas after cranial radiotherapy for childhood cancers
Source: Neurooncol Adv. 2022 Oct 3;4(1):vdac159. doi: 10.1093/noajnl/vdac159 (PMC9639354; doi:10.1093/noajnl/vdac159)
Supplement: vdac159_suppl_Supplementary_Data [file vdac159_suppl_supplementary_data.docx]

**Supplemental Figure 1.** RIG cumulative incidence over time (Cohort 1a)

**Supplemental Figure 2.** A) Percentage of all patients by year of original diagnosis who eventually developed RIG. B) RIG diagnoses as a percentage of CNS tumor patients by year.
