# Supplementary figures and images for: Population-based analysis of radiation-induced gliomas after cranial radiotherapy for childhood cancers
Source: Neurooncol Adv. 2022 Oct 3;4(1):vdac159. doi: 10.1093/noajnl/vdac159 (PMC9639354; doi:10.1093/noajnl/vdac159)

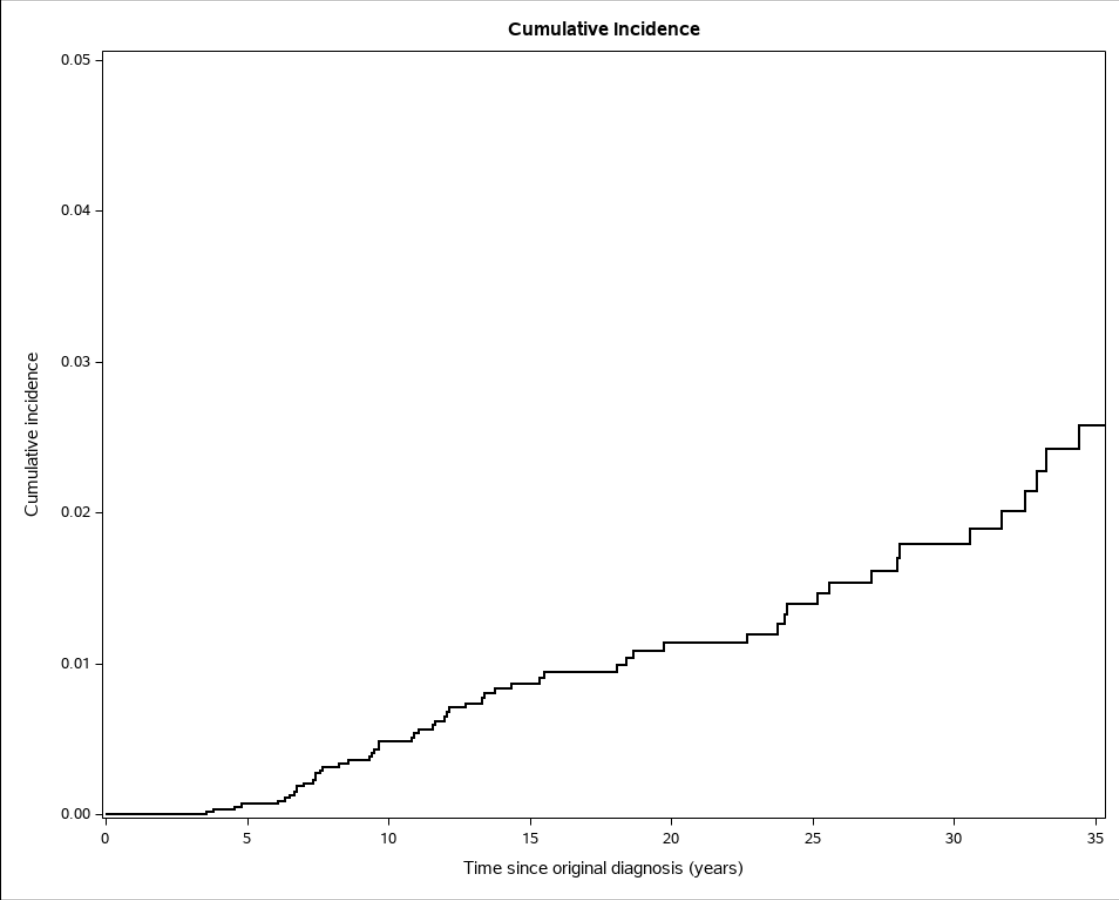

**A** Cohorts 1a and 1b

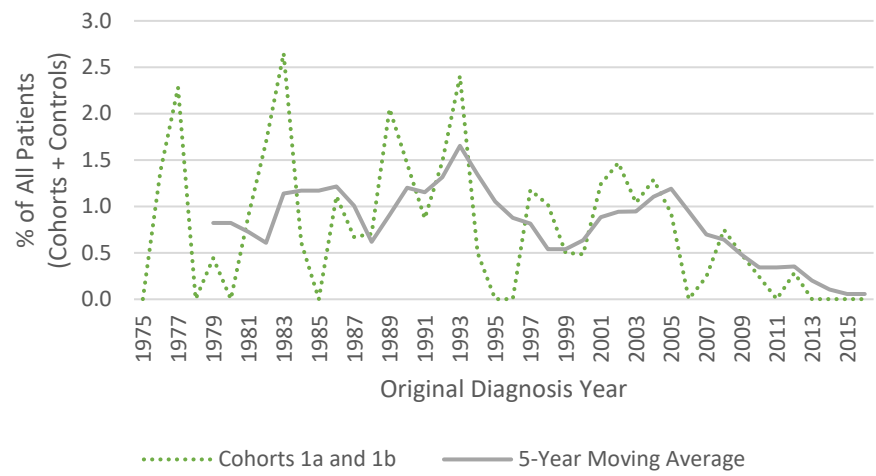

**B** RIG Diagnoses (Cohorts 1a and 1b)

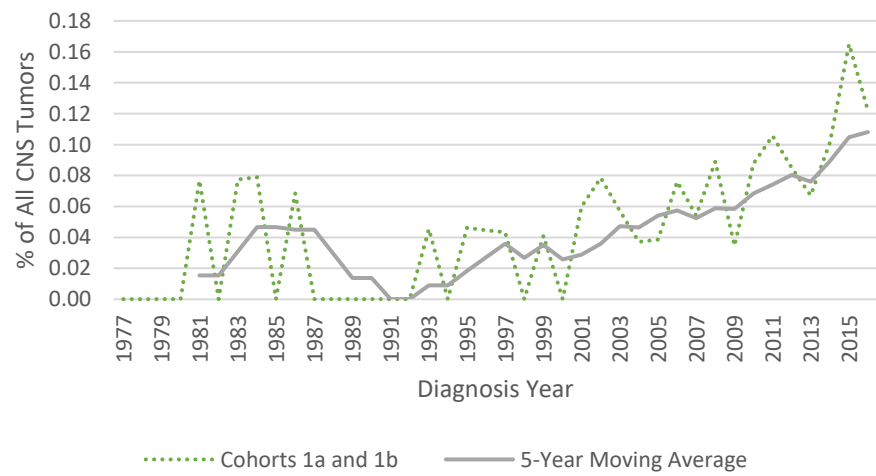

Supplement: vdac159_suppl_Supplementary_Figures [file vdac159_suppl_supplementary_figures.pdf]
